# Genome‐wide Interaction and Stratified Study with Smoking Identifies Association of *APAF1* and *MIXL1/LIN9* with Alzheimer’s Disease

**DOI:** 10.1002/alz.091434

**Published:** 2025-01-03

**Authors:** Ryan Dacey, Xudong Han, Mackenzie R Moore, Jaeyoon Chung, Shruti Durape, Max Rosenthaler, Madeline Uretsky, Bobak Abdolmohammadi, Annie J. Lee, Adam M. Brickman, Timothy J. Hohman, Michael L. Cuccaro, David A. Bennett, Paul K. Crane, M. Ilyas Kamboh, Walter A. Kukull, Rhoda Au, Jonathan L. Haines, Margaret A. Pericak‐Vance, Gerard D. Schellenberg, Richard Mayeux, Kathryn L. Lunetta, Lindsay A. Farrer, Jesse Mez

**Affiliations:** ^1^ Framingham Heart Study, Boston University Chobanian & Avedisian School of Medicine, Boston, MA USA; ^2^ Department of Medicine (Biomedical Genetics), Boston University Chobanian & Avedisian School of Medicine, Boston, MA USA; ^3^ Department of Neurology, University of Washington, Seattle, WA USA; ^4^ Boston University Alzheimer’s Disease Research Center, Boston University Chobanian & Avedisian School of Medicine, Boston, MA USA; ^5^ Department of Neurology, Boston University Chobanian & Avedisian School of Medicine, Boston, MA USA; ^6^ Department of Neurology, Columbia University, New York, NY USA; ^7^ Vanderbilt Genetics Institute, Vanderbilt University Medical Center, Nashville, TN USA; ^8^ Vanderbilt Memory and Alzheimer’s Center, Vanderbilt University Medical Center, Nashville, TN USA; ^9^ The John P. Hussman Institute for Human Genomics, University of Miami, Miami, FL USA; ^10^ Department of Neurological Sciences, Rush Medical College, Chicago, IL USA; ^11^ Department of Medicine, University of Washington, Seattle, WA USA; ^12^ Department of Human Genetics, University of Pittsburgh, Pittsburgh, PA USA; ^13^ Department of Epidemiology, Boston University School of Public Health, Boston, MA USA; ^14^ Boston University Chobanian & Avedisian School of Medicine and School of Public Health, Boston, MA USA; ^15^ Department of Anatomy & Neurobiology, Boston University Chobanian & Avedisian School of Medicine, Boston, MA USA; ^16^ Department of Epidemiology and Biostatistics, Case Western Reserve University, Cleveland, OH USA; ^17^ John P. Hussman Institute for Human Genomics, Miami, FL USA; ^18^ Department of Pathology and Laboratory Medicine, University of Pennsylvania, Philadelphia, PA USA; ^19^ Department of Neurology, Vagelos College of Physicians & Surgeons, Columbia University, New York, NY USA; ^20^ Department of Biostatistics, Boston University School of Public Health, Boston, MA USA; ^21^ Department of Ophthalmology, Boston University Chobanian & Avedisian School of Medicine, Boston, MA USA; ^22^ Framingham Heart Study, Boston, MA USA

## Abstract

**Background:**

Alzheimer’s disease (AD) has both genetic and environmental risk factors. Gene‐environment interaction may help explain some missing heritability. There is strong evidence for cigarette smoking as a risk factor for AD. To identify genetic‐smoking‐related associations with AD, we conducted a genome‐wide association study (GWAS) assessing a SNP‐smoking interaction and stratified analysis by smoking status.

**Method:**

Lifetime smoking data were available and analyzed among 22,030 non‐Hispanic White (NHW; 8,232 cases; 13,798 controls) and 3,126 African American (AFA; 921 cases; 2,205 controls) participants from the AD Genetic Consortium and the Framingham Heart Study. “Ever smoking” status was considered as a dichotomous exposure, defined by current smoking status or past history of smoking. Across 35 datasets, we conducted GWAS with two approaches: inclusion of a SNP‐by‐smoking interaction term and stratification by smoking status (12,080 smokers, 13,428 non‐smokers). MAGEE was used to estimate SNP‐by‐smoking interaction effects and SAIGE was used to estimate SNP effects in stratified analysis. Age, sex, and principal components for population structure were included as covariates. METAL was used for inverse‐variance weighted meta‐analysis across datasets to estimate within‐ and cross‐ancestry effects.

**Result:**

The stratified analysis identified a genome‐wide significant association among smokers in *APAF1* on chromosome 12 (top SNP: rs12368451; smokers: MAF = 0.44, p = 2.2 × 10^‐8^, OR = 1.20; non‐smokers: MAF = 0.44, p = 0.97, OR = 1.00). Effects were present in both ancestry groups (NHW: MAF = 0.45, p = 6.1 × 10^‐6^, OR = 1.16; AFA: MAF = 0.35, p = 6.6 × 10^‐5^, OR = 1.46). *APAF1* has been linked to gene‐smoking interaction for non‐AD related outcomes. A neighboring gene, *ANKS1B*, is highly expressed in the brain, interacts with amyloid‐b precursor protein, and has shown GWAS signals for smoking initiation and cognitive ability. We also identified a genome‐wide significant SNP‐by‐smoking interaction in the *MIXL1/LIN9* region on chromosome 1 (top SNP: rs1091961, MAF = 0.35, p = 4.9 × 10^‐8^, β_SNP*smoking_ = 0.24; smokers: OR = 1.12, p = 0.0006; non‐smokers: OR = 0.89, p = 0.0001). Within *LIN9*, several SNPs in linkage disequilibrium with rs1091961 have shown sub‐genome wide association with nicotine dependence.

**Conclusion:**

In this gene‐smoking interaction and smoking‐stratified GWAS of AD, we identified two promising loci. These findings highlight the strength of utilizing cross‐ancestry datasets and considering both genetic and environmental factors together towards a personalized medicine approach to AD.

